# Assessing the Role of Carbonyl Adducts, Particularly Malondialdehyde Adducts, in the Development of Dermis Yellowing Occurring during Skin Photoaging

**DOI:** 10.3390/life12030403

**Published:** 2022-03-10

**Authors:** Hélène Zucchi, Hervé Pageon, Daniel Asselineau, Marion Ghibaudo, Inês Sequeira, Sarah Girardeau-Hubert

**Affiliations:** 1 L’Oréal Research and Innovation, 1 Avenue E. Schueller, 93600 Aulnay-sous-Bois, France; helene.zucchi@rd.loreal.com (H.Z.); daniel.asselineau@wanadoo.fr (D.A.); marion.ghibaudo@club-internet.fr (M.G.); i.sequeira@qmul.ac.uk (I.S.); sarah.hubert@rd.loreal.com (S.G.-H.); 2 GenSight Biologics, 75012 Paris, France; 3 Barts and the London School of Medicine and Dentistry, Queen Mary University of London, London E1 4NS, UK

**Keywords:** yellowish, dermis, photoaging, carbonylation, malondialdehyde, 4-hydroxynonenal, acrolein

## Abstract

Solar elastosis is associated with a diffuse yellow hue of the skin. Photoaging is related to lipid peroxidation leading to the formation of carbonyl groups. Protein carbonylation can occur by addition of reactive aldehydes, such as malondialdehyde (MDA), 4-hydroxy-nonenal (4-HNE), and acrolein. All the proteins concerned with this modification, and the biological consequences of adduct formation, are not completely identified. The link between yellowish skin and dermal carbonylated proteins induced by aldehyde adducts was investigated. The study was carried out on ex vivo skin samples from sun-exposed or sun-protected areas and on in vitro dermal equivalent models incubated with 5 mM MDA, 4-HNE, or acrolein. The yellow color and the level of MDA, 4-HNE, and acrolein adducts were evaluated. Yellowish color differences were detected in the dermis of sun-exposed skin compared to sun-protected skin and in in vitro models following addition of MDA, 4-HNE, or acrolein. The yellowing was correlated with the carbonyl adducts increasing in the dermis and in in vitro models incubated with aldehydes. The stronger yellowing seemed to be mediated more by MDA than 4-HNE and acrolein. These observations suggest that dermal carbonylation especially induced by MDA result in the yellow hue of dermis and is involved, in part, in the yellowing observed during skin photoaging.

## 1. Introduction

**Figure 1:**
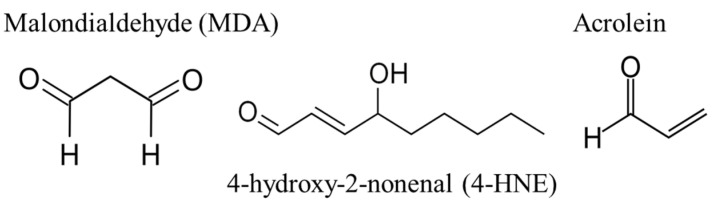
Structures of malondialdehyde (MDA), 4-hydroxy-2-nonenal (4-HNE), and acrolein.

Skin photoaging is an accelerated aging mechanism that results from exposure to ultraviolet (UV) radiation leading to hypertrophic skin, deep wrinkles, loss of skin elasticity, a leathery skin, and a yellow discoloration [1]. This skin yellowness is described on photodamaged skin sites as well as on several skin disorders, involving alteration of the elastic tissue [2]. These clinical signs are initiated within the dermis, which is the main target of UVAs that induce oxidative damage and collagen degradation. A hallmark of protein oxidative stress and aging is carbonylation [3], which is achieved by the formation of aldehydes derived from lipid peroxidation and considered as a major contributor of protein oxidative damage [4]. During lipid peroxidation, reactive oxygen species can oxidize membrane lipids, generating lipid hydroperoxides and many aldehydes such as malondialdehyde (MDA), 4-hydroxy-nonenal (4-HNE), and acrolein (Figure 1). These aldehydes can form into carbonyl groups on lysine, histidine, and cysteine of proteins resulting in modified proteins called adducts or ALE (Advanced Lipoxidation End-products) [5]. A previous study showed carbonyl modification of dermal extracellular matrix proteins to be correlated with the yellowish skin in Asian (Japanese) sun-exposed facial skin, associated with photoaging and solar elastosis [6]. In vitro long-term treatment with 4-HNE and acrolein lead to the appearance of the yellowish dermal changes, suggesting that carbonyl modification is involved in this process. Furthermore, 4-HNE contributes to fibroblast senescence upon UV radiation [7,8]. However, MDA adducts also accumulate during aging and after solar UV radiation [9], but little is known about how MDA affects the skin’s yellowish color change.

In this study, we aim to understand the role of MDA in skin yellowing occurring with photoaging by analyzing the dermis of sun-exposed skin and sun-protected skin, through colorimetric parameters, protein carbonyl detection, and by testing in vitro the effect of MDA in dermis skin yellowing.

## 2. Materials and Methods

### 2.1. Human Skin Samples

Twenty-three skin samples from adult women with Caucasian skin type (phototype I–III) were collected. Skin explants, collected from healthy subjects undergoing reconstructive or aesthetical surgery, were obtained from Icelltis (Toulouse, France) and Alphenyx (Marseille, France) under the authorizations delivered by the French Ministry of Research with the approval of the French Ethical Committee. Written informed consent was obtained from all donors. Sun-protected skin samples were obtained from breast surgery tissue (*n* = 6, age range 53 to 68 years old). Sun-exposed skin samples were obtained from face-lift (cheek) surgery tissue (*n* = 17, age range 57 to 75 years old).

### 2.2. In Vitro Models: Acellular Dermis and Carbonylation

Normal human skin fibroblasts were isolated from adult Caucasian donors undergoing plastic mammary surgery. The dermal equivalent was produced after contraction at 37 °C of a mixture of bovine type I collagen (Symatèse, Lyon, France) and fibroblasts, as previously described [10]. After dermal equivalent contraction, fibroblasts were slayed by osmotic shock with water followed by extensive washing with water and kept in PBS (Phosphate-Buffered Saline). To induce protein carbonylation, acellular dermis were incubated with 5 mM of malonedialdehyde (MDA) from Merck (Darmstadt, Germany), 4-hydroxy-nonenal (4-HNE) from Cayman Chemical (Ann Arbor, MI, USA), or acrolein from Sigma-Aldrich (Saint Louis, MO, USA) in PBS for 3 weeks at 37 °C. The solutions were renewed twice a week. As negative controls, some samples were incubated with PBS only.

### 2.3. Histological Staining

Human skin samples were fixed in neutral formalin then embedded in paraffin. We stained 5 µm paraffin sections with orcein for elastic fiber detection and with sirius red for collagen fiber visualization. Using Histolab software (Microvision Instrument, Lisses, France), the percentage of dermal elastin (including elastotic tissue) and collagen surface were quantified.

### 2.4. Immunostaining

Immunofluorescence was performed on 5 µm thick paraffin sections using specific antibodies: rabbit anti-human MDA (ab6463, Abcam, Cambridge, UK), rabbit anti-human 4-HNE (ab46545, Abcam, Cambridge, UK), and mouse anti-human acrolein clone 5F6 (ab48501, Abcam, Cambridge, UK). Briefly, after a heat-mediated antigen retrieval step, using citrate buffer pH 6, samples were blocked using 10% goat serum (X0907, Agilent Technologies, Les Ulis, France) for 30 min at room temperature. Then, sections were incubated with the primary antibody’s solutions at 1:200 overnight at 4 °C. Primary antibodies were detected by a goat anti-mouse (A-11029, ThermoFischer Scientific, Waltham, MA, USA) or a goat anti-rabbit (A-21428, ThermoFischer Scientific, Waltham, MA, USA) conjugated to Alexa fluor 488 at 1:500. Stained tissue sections were mounted using the Prolong^TM^ Gold mounting medium with Dapi (P36941, Invitrogen, Waltham, MA, USA). Immunostainings were analyzed with a Leica microscope coupled with a camera Retiga 2000R (QImaging, Surrey, BC, Canada). Histolab software (Microvision Instrument, Lisses, France) was used for quantitative image evaluation. The level of labeled protein expression was quantified in the dermis as fluorescent integrated emission (surface * intensity). Results were expressed as percentages of areas of the measured dermis.

### 2.5. Colorimetric Measurements of Skin Samples and In Vitro Dermis: Individual Typology Angle (ITA°) Determination and b* Parameter Measurement

**Figure 2:**
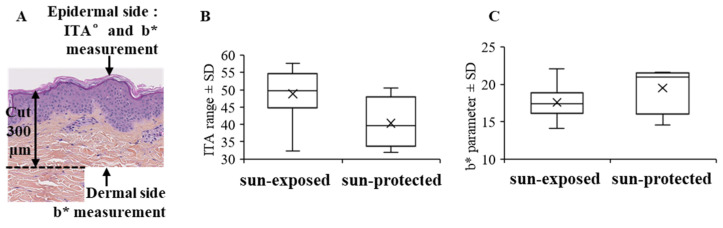
Ex vivo human skin colorimetric measurement procedure (**A**), ITA (**B**), and b* parameter (**C**) measurements from epidermal side of sun-exposed and sun-protected skin; no significant differences were observed for ITA and b* parameter for epidermis. ITA, individual typology angle.

Color was measured by a spectro-colorimeter (Digieye, VeriVide Limited, Leicester, UK) to obtain the color coordinates L*, a*, b* which characterize colors: lightness L* derives from the luminance of the surface and the two parameters a* and b* express the difference in color compared to a gray surface of the same lightness. For the ex vivo human skin sample, the individual typology angle (ITA) reflecting the skin color for each skin sample was calculated based on these spectrophotometric measurements. ITA allowed us to classify skin type color and to select skin samples with a similar skin type (Figure 2A) [11]. The b* parameter that represents the yellow-blue component was analyzed. To avoid the potential effects of the keratin coloration after reaction to pollutants or the presence of melanin in the colorimetric data, the b* parameter was first measured from the epidermal side to determine if this parameter was similar for the epidermis between skin samples (Figure 2A). For the dermis, colorimetric measurements were performed from the dermal skin (Figure 2A). The skin sample was cut to a depth of 300 µm, and measures were carried out on the upper dermis (above 300 µm) after turning it over. Concerning in vitro models, measures were performed directly on all the samples.

### 2.6. Dermal Protein Oxidation Detection

After dermal-epidermal separation, the overall content of carbonyl proteins (Oxi-Proteome) in the dermal protein extracts was obtained from ex vivo sun-protected versus sun-exposed aged samples. These analyses and quantification were performed by OxiProteomics (Creteil, France).

### 2.7. Statistics

The Wilcoxon signed-rank test was used to compare the distinct groups and the *p*-values and effect sizes estimated. *p*-values < 0.05 were considered significant.

## 3. Results

### 3.1. Yellowing of the Dermis Is Increased on Photo-Exposed Skin

**Figure 3:**
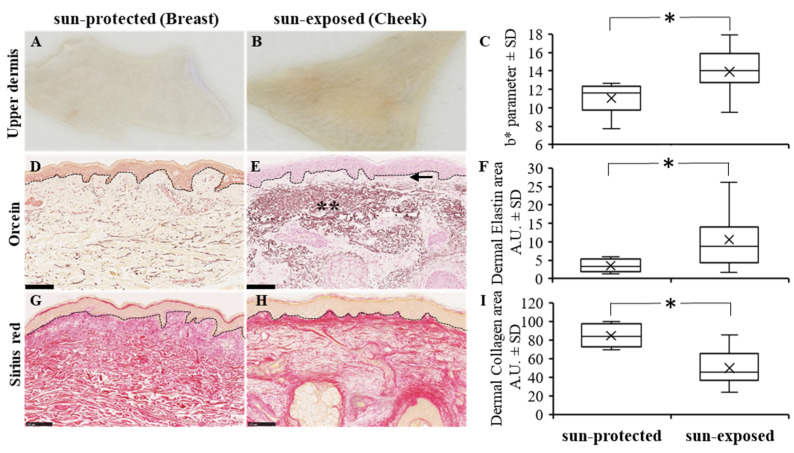
Ex vivo human dermis skin (**A**,**B**), b* parameter measurement (**C**), and orcein (**D**,**E**) and sirius red (**G**,**H**) histological stains from sun-protected (**A**,**D**,**G**) and sun-exposed (**B**,**E**,**H**) human skin (scale bar = 100 µm). Detection of b* parameter, elastin, and collagen in dermis were increased in sun-exposed skin as compared to sun-protected skin as reported in box plot (**C**,**F**,**I**, respectively) (* *p* < 0.05); arrow and ** indicate, respectively, the grenz zone and amorphous elastic aggregate both characteristic of elastosis area in sun-exposed dermis.

First, we choose skin samples sun-exposed and sun-protected with a similar phototype from I to III. In order to avoid any differences in the b* parameter between skin samples from a pigmented epidermis, the individual typology angle (ITA) reflecting the skin color for each skin sample was calculated [11], and no significant differences were detected between sun-exposed and sun-protected skin samples (Figure 2B). Second, to rule out that epidermis staining could affect the colorimetric measurements, we compared the b* parameter between skin samples measured from the epidermal side and we did not find any significant difference between sun-exposed and sun-protected skin samples (Figure 2C). Therefore, in our study, we measured the parameter b* from the dermal side of the samples of light skin (Figure 3A,B) in order to avoid the possible staining of the epidermis and to only consider the yellowness of the dermis. Dermis from sun-exposed skin presented a higher yellowish appearance (b* parameter, *p* < 0.05) than sun-protected skin (Figure 2C).

### 3.2. Collagen and Elastic Networks Are Altered in Photoexposed Skin

It is well known that with photoexposure the elastic and collagen networks are modified. It has been previously reported that extracellular matrix composition and architecture were altered with aging [12,13]. Skin exhibits distinct features with high UV exposure, and in photo-exposed skin, it has been observed that an abnormal accumulation of elastotic material and increased collagen degradation could occur [14,15]. We aimed to characterize the dermis for elastic component and for collagen fibers using orcein and sirius red staining, respectively. In our skin samples, analyses, and quantification of the dermis elastic network, we showed that the elastin fibers in sun-protected skin samples were mostly thin and dispersed throughout the dermis (Figure 3D), while sun-exposed skin dermis presented a moderate to severe elastosis with increased thickness of elastic fibers (Figure 3E). Interestingly, on sun-exposed skin the elastic fibers appeared as wide and heavy stained bands particularly in the reticular dermis, while the thin region closer to the epidermis was mostly an unstained zone, called the grenz zone [14] (Figure 3E, shown with double asterisk and arrow, respectively). The amount of elastin was significantly higher in sun-exposed skin than in sun-protected skin (*p* < 0.05) (Figure 3F). The observed accumulation of elastotic material was associated with a degeneration of the adjacent collagen network. Indeed, we observed a significant decrease in the level of collagen fibers in the sun-exposed as compared to sun-protected dermis (*p* < 0.05) (Figure 3G–I).

### 3.3. Dermal Protein Oxidation Increases with Photoexposure

**Figure 4:**
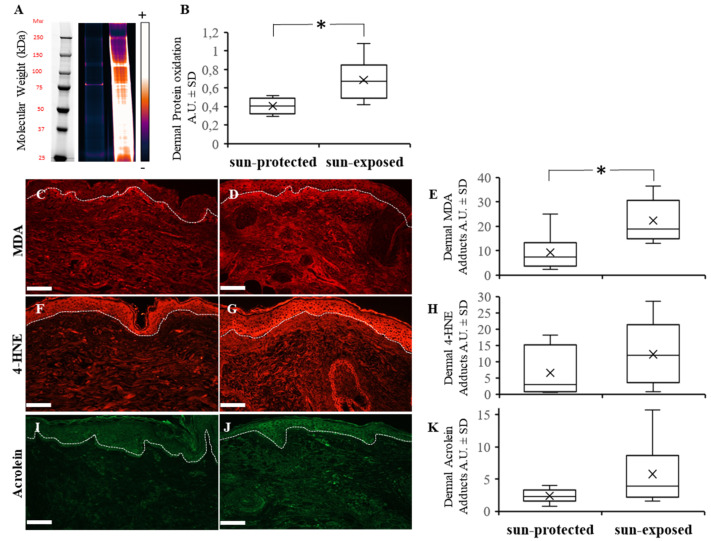
Carbonylation measurements: Oxiproteome (**A**) and malonedialdehyde—MDA (**C**,**D**), 4-Hydroxy-nonenal—4-HNE (**F**,**G**) and acrolein (**I**,**J**) adducts immunostainings from sun-protected (**C**,**F**,**I**) and sun-exposed (**D**,**G**,**J**) human skin (Scale bar = 50 µm). Quantification of dermal protein oxidation from Oxiproteome, dermal MDA, 4-HNE and acrolein were increased in sun-exposed skin as reported in box plot (**B**,**E**,**H**,**K**, respectively) (* *p* < 0.05).

The proteome is a target for damage by UV-induced reactive oxidative species. Immunoblot analysis of carbonyl residues (Oxiproteome) in tissue extracts from dermis showed that chronic UV exposure in sun-exposed skin induced the accumulation of protein oxidation in human skin (*p* < 0.05) (Figure 4A,B).

### 3.4. Oxidation Adducts and Especially Malonedialdehyde Adducts Increase in Photoexposed Skin

Analysis of the levels of MDA, 4-HNE, and acrolein adducts assessed by immunofluorescence showed a higher accumulation of MDA adducts (*p* < 0.05), but not a significant accumulation of 4-HNE and acrolein adducts in sun-exposed dermis when compared to sun-protected dermis (Figure 4C–K). These results indicate that lipoxidation products induced by photoexposure are likely involved in the accumulation of damaged proteins.

### 3.5. Yellowing of the Equivalent Dermis Increases in the Presence of Oxidation Adducts

**Figure 5:**
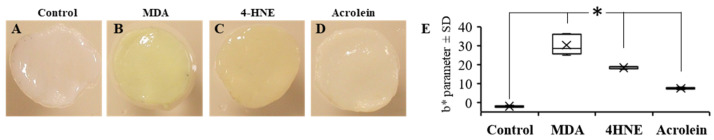
Yellowish coloration after treatment with 5 mM MDA (**B**), 4-HNE (**C**) and acrolein (**D**) compared to control without treatment (**A**) and b* parameter measurement (**E**) of in vitro dermal equivalents (* *p* < 0.05). Note that the intensity of yellowing was higher in the order MDA > 4-HNE > acrolein > control.

Our previous results obtained with human skin suggest that with photo-exposure the accumulation of oxidized protein and yellowing may be related. Therefore, to assess this hypothesis, we conducted in vitro validation studies of dermis yellowing using dermal equivalent models. The dermal equivalent models, composed of type I collagen, were treated with MDA, 4-HNE, or acrolein at the same concentration to confirm the link between carbonyl modification of collagen and yellowish skin macroscopic color (Figure 5A–D). Our results showed an increase in the b* parameter (yellowish color) not only in incubation of dermal equivalents with 4-HNE and acrolein (b* parameter = 18 ± 0.4 and 7 ± 0.3, respectively) as seen before [6], but an even more significant b* parameter increase when treated with MDA atthe same concentration (b* parameter = 30 ± 3) (Figure 5E). This clearly demonstrates that incubation with the lipid peroxidation product MDA resulted in a stronger modification of collagens protein and a yellowing of the dermal equivalent. These results support our in vivo observations suggesting that MDA is an important effector of dermal yellowing. 

## 4. Discussion

Skin photoaging is an accelerated aging mechanism that results from exposure to ultraviolet rays combined with chronological aging. The clinical features of photoaging can be atrophic skin with mild wrinkles, abnormal epidermal proliferation and depigmentation disorders or hypertrophic skin, with deeper wrinkles, a leathery skin, and a yellow discoloration [1,12,13]. Many of these clinical signs are initiated within dermis, the main target of UVA’s radiation. Indeed, actinic elastosis, a hallmark of photoaging, show profound changes in dermal proteins as an accumulation of elastotic material composed of abnormal elastin or collagens and proteoglycans degradation [12,13,14,15,16,17,18]. 

It has been suggested that UV irradiation induces the formation of Reactive Oxygen Species (ROS) in cutaneous tissues, which are considered the main contributors to photoaging [16,19]. ROS can have deleterious effects on proteins directly or indirectly by secondary messengers, such as during lipid peroxidation [3]. Recent studies suggested that protein carbonylation formed from aldehydes derived from lipid peroxidation is a major contributor of oxidative damage of proteins [20,21].

The present work attempted to characterize sun-exposed skin compared to sun-protected skin from old Caucasian women, considering the skin yellowing as a perceptible feature of photoaging. We focused on the dermis and analyzed their colorimetric parameters and protein carbonyl expressions. The first results showed that the dermis from sun-exposed skin presented a more yellowish appearance, as compared to sun-protected skin. This result agreed with previous results from Oshima et al. [22] that described an increase in the b* value of colorimetry of facial skin, with age, in Japanese women. In 2011, Ogura et al. [6] provided evidence that the yellowish change in the dermis is one of the main factors that lead to the yellowish appearance of the skin in elderly Japanese. Here, we demonstrated that a yellowish skin dermis discoloration can also appear on photo-aged Caucasian skins.

Carbonyl groups may be introduced into proteins by addiction of reactive aldehydes derived from the oxidation of polyunsaturated fatty acids (PUFAs) [4]. Aldehydes, mainly MDA, 4-HNE, or acrolein, were considered as second messengers of free radicals; physiologically highly active, they were found involved in numerous diseases’ progression [23]. They could react with cysteine, histidine, and lysine residues in proteins [3,24], producing Advanced Lipoxidation End products (ALEs). In the skin, proteins from the extracellular matrix were altered by 4-HNE or MDA adducts [23]. Ogura et al. [5] and Tanaka et al. [25] showed that 4-HNE adducts were detected in actinic elastosis skin. Larroque-Cardoso et al. demonstrated an elastin modification by 4-HNE in hairless mice [7]. They showed a strong increase in 4-HNE adducts after UVA irradiation and a co-localization between 4-HNE and acrolein adducts with elastin. In our study, we also demonstrated a trend accumulation of 4-HNE and acrolein adducts in the dermal extracellular matrix from sun-exposed skin. Additionally, we found a stronger accumulation of MDA adducts in sun-exposed, as compared to sun-protected skin. MDA adducts formation has been previously observed in cultured skin keratinocytes, murine and human skin exposed to solar UV and in skin cancer tissue [26]. 4-HNE and MDA were known to cause damage and cross-linking of collagen [27] and were involved in the formation of highly cross-linked fluorescent and pigmented protein aggregates referred to as lipofuscin [28,29]. However, even if an accumulation of adducts was detected in higher level in sun-exposed skin compared to sun-protected skin, a clear correlation with the yellowish hue has still not been completely established.

**Figure 6:**
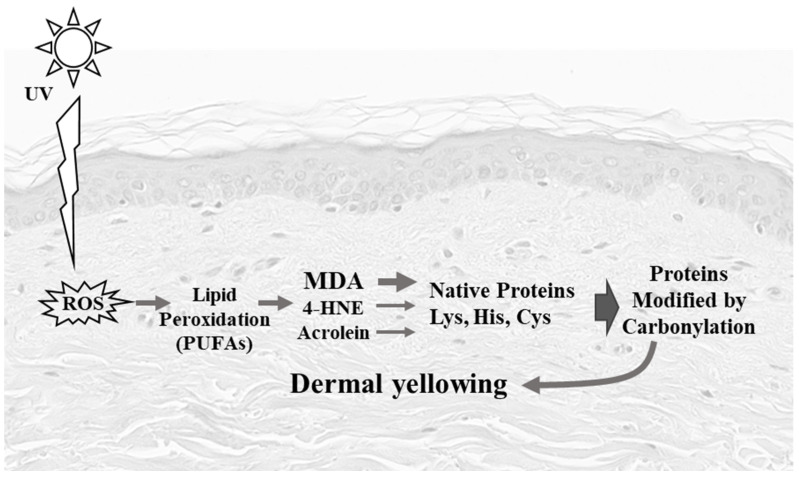
Schematic representation of yellowing of the dermis by carbonyl adducts: photoexposure (especially UVA radiation), induces free radicals which can directly attack polyunsaturates fatty acids (PUFAS) in membranes and initiate lipid peroxidation. PUFAs are degraded to a variety of products. Some of them such as aldehydes (e.g., MDA, 4-HNE, acrolein) are very reactive and can damage proteins and cause the yellowing observed in our study.

In this study, we observed a higher accumulation of MDA adducts in sun-exposed skin. To highlight the role of MDA in the formation of yellowing skin, we carried out in vitro experiments using dermis equivalents, composed of type I collagen, treated with aldehydes derived from lipid peroxidation. We observed a clear yellowish color modification of acellular equivalent dermis incubated with a non-physiological concentration of MDA, 4-HNE, or acrolein to accelerate the process of carbonylation. At the same concentration, MDA is the most efficient agent to produce yellow aggregates when compared to 4-HNE and acrolein. These results support our in vivo observations suggesting that collagen is an important target of the MDA as summarized in Figure 6.

It would be interesting in future work to study the evolution of the b* parameter in photo-exposed versus non-photo-exposed skin, from the same donor and/or from a younger donor. Furthermore, we could consider studying the synergistic in vitro effect of the various adducts in the phenomenon of photoaging in our models to mimic the photoaging phenomenon occurring in the skin.

This study highlights the significant role of MDA in the development of the yellowish color change in sun-exposed skin, contributing to the skin damage observed during photoaging. This is of particular importance for elderly people, particularly of Asian skin type, that tend to show a significant yellowish skin color change with photoaging. The use of suitable sunscreen with or without treatment with compounds that can reduce the formation of or trap carbonyl proteins such as MDA, e.g., carnosine [30,31], could limit the deleterious effects of adducts as well as the yellowing of the skin observed during photo-exposure. Understanding the biological mechanisms behind skin yellowness, including how MDA and other adducts such as 4-HNE and acrolein induce carbonyl modification leading the yellowish changes in skin, could provide valuable insights to prevent photoaging and help to develop new strategies to prevent and reduce skin yellowness.

## Data Availability

The data used to support the findings of this study are included within the article. The detail of the data presented in this study are available on request from the corresponding author.
